# PopHumanScan: the online catalog of human genome adaptation

**DOI:** 10.1093/nar/gky959

**Published:** 2018-10-18

**Authors:** Jesús Murga-Moreno, Marta Coronado-Zamora, Alejandra Bodelón, Antonio Barbadilla, Sònia Casillas

**Affiliations:** Institut de Biotecnologia i de Biomedicina and Departament de Genètica i de Microbiologia, Universitat Autònoma de Barcelona, 08193 Bellaterra, Barcelona, Spain

## Abstract

Since the migrations that led humans to colonize Earth, our species has faced frequent adaptive challenges that have left signatures in the landscape of genetic variation and that we can identify in our today’s genomes. Here, we (i) perform an outlier approach on eight different population genetic statistics for 22 non-admixed human populations of the Phase III of the 1000 Genomes Project to detect selective sweeps at different historical ages, as well as events of recurrent positive selection in the human lineage; and (ii) create *PopHumanScan*, an online catalog that compiles and annotates all candidate regions under selection to facilitate their validation and thoroughly analysis. Well-known examples of human genetic adaptation published elsewhere are included in the catalog, as well as hundreds of other attractive candidates that will require further investigation. Designed as a collaborative database, PopHumanScan aims to become a central repository to share information, guide future studies and help advance our understanding of how selection has modeled our genomes as a response to changes in the environment or lifestyle of human populations. PopHumanScan is open and freely available at https://pophumanscan.uab.cat.

## INTRODUCTION

Since the split with chimpanzees, and especially since the migrations that led humans to colonize almost every single place on Earth, our species has faced frequent environmental and social changes that have shaped the variation patterns of our genomes through the action of natural selection (1). These environmental challenges include, for example, extreme cold temperatures in much of the Americas and Eurasia during the last ice age, limiting exposure to sunlight as we moved to higher latitudes or contact with new pathogens. Part of the incorporated genetic innovations may have been introgressed from archaic hominins that left Africa before us, including Neanderthals and Denisovans, with whom we encountered and interbred before they got extinct. Around 1–6% of any modern non-African human genome can be traced back to the genomes of these archaic populations (2). Another dramatic change occurred within the past 10 000 years coinciding with the transition from a hunting-gathering lifestyle to farming. Selection pressures for adapting to large settlements and new diets favored genetic variants associated with innate immune response, fatty acid metabolic efficiency, and lactose tolerance, among others (3).

These selection pressures left signatures in the landscape of genetic variation that can be identified in our today’s genomes (4). Starting from single-locus studies to the first large-scale catalogs of genetic variation (5–8), dozens of targets of positive selection have been identified, providing important insights into recent human evolutionary history (3,9,10). Even though genome-wide HapMap genotyping data is able to disentangle the effects of demography and selection better than single-locus approaches, it still has the problem of ascertainment bias, which may alter the site frequency spectrum (SFS) of analyzed single nucleotide polymorphisms (SNPs) (11). The availability of the most comprehensive worldwide nucleotide variation dataset so far from the 1000 Genomes Project (1000GP) (12,13), based on whole-genome re-sequencing, provides the human lineage with an abundant, ascertained variation dataset on which to test molecular population genetics hypotheses and eventually pinpoint targets of positive selection in one or more human populations that escape from the background evolutionary dynamics of genetic variation (14).

To gain deeper understanding of how environmental and social challenges have shaped our genomes through the action of natural selection, here we (i) perform a genome-wide scan of selection on the latest version of the 1000GP data by surveying distinctive signatures of genomic variation left by different selective events, and (ii) create an online catalog of all candidate genomic regions under selection to facilitate their validation and thorough analysis. As far as we are concerned, dbPSHP (15) is the only previous online database that compiles putative positively selected loci in human evolution. In their case, regions were extracted from curated publications based on genotyping data—instead of whole-genome re-sequencing data—of the HapMap III (8) and the 1000GP Pilot 1 (12), and the last update is reported as far as May 2014. On the other hand, after the publication of the 1000 Genomes Selection Browser 1.0 (16), Pybus *et al.* developed a machine-learning framework—Hierarchical Boosting—that combines the results of multiple tests for detecting positive selection to classify genomic regions into different selection regimes (17). They analyzed within-species polymorphism data for three populations of the 1000GP Phase I (12), and the resulting scores were made available as UCSC Hub Tracks. Here, we perform an outlier approach on the greatest number of population genetic statistics and sampled populations available so far. This genome-wide scan of selection is able to detect sweeps at different historical ages, as well as evidence of recurrent selection in the human lineage since the split between our species and chimpanzees. Results have been made available in a collaborative, online database, *PopHumanScan*, which is aimed at compiling and annotating adaptation events along the human evolutionary history. Well-known examples of human genetic adaptation published elsewhere are included in the catalog, as well as hundreds of other attractive candidates that will require a more thoroughly analysis. PopHumanScan graphically represents each signature of selection within the empirical distributions of the corresponding DNA diversity statistic across populations. It also provides structural and functional annotations of the region, links to external databases and cross-references to 268 publications.

## POPHUMANSCAN ANALYSIS PIPELINE

**Figure 1.**
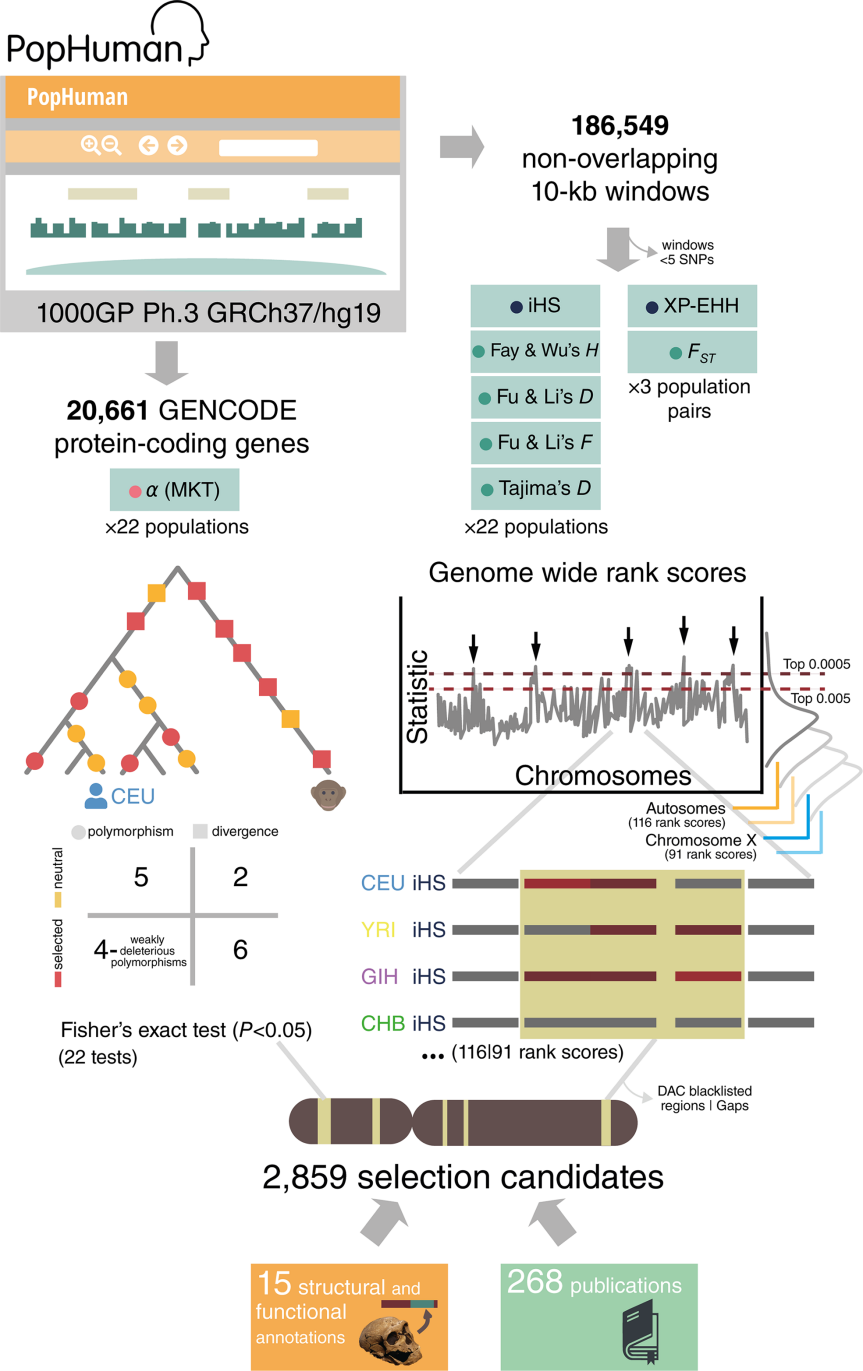
PopHumanScan pipeline. Starting from population genomic data retrieved from PopHuman, 8 different neutrality tests are analyzed in 22 non-admixed human populations (or 3 population pairs). Tests are color-coded depending on the type of signature they are able to detect: 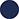
*Linkage Disequilibrium* (*LD*), 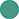
*Site Frequency Spectrum (SFS)* and 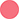
*Protein Changes*. The significance of each test is assessed either with a Fisher's exact test or a rank score, for each of the 22 populations (or 3 population pairs) independently, and independently for autosomes and the X chromosome. Finally, candidate regions under selection are structurally and functionally annotated, and cross-referenced with 268 publications.

We have designed and implemented a custom pipeline (Figure 1) to perform a genome-wide scan of selection. Specifically, the pipeline processes eight different neutrality tests calculated either in sliding windows along the genome or for each protein-coding gene, for 22 non-admixed human populations. The genomic regions identified should show signatures that are compatible with natural selection having driven the evolution of the region at one or different timescales, from recent selective sweeps to recurrent selection since the split between our species and chimpanzees. These candidate regions under selection are further characterized with structural and functional annotations of that particular region. Furthermore, 268 articles reporting evidences of natural selection in genomic regions and genes using different statistical methods have been manually curated and cross-referenced to the candidate regions detected with our pipeline.

### Pre-processing of the PopHuman data

Population genomic data were retrieved from PopHuman (18) for 22 non-admixed populations of the Phase III of the 1000GP (13) (Supplementary Table S1), mapped to GRCh37/hg19. Specifically, values for seven different neutrality tests have been obtained for each population in 186 549 10-kb non-overlapping sliding windows along the autosomes and the X chromosome (Figure 1). In addition, the McDonald and Kreitman test (MKT) (19), as well as the proportion of substitutions that are adaptive (*α*) (20,21), were calculated on the protein-coding genes overlapping the candidate regions under selection identified with the other seven statistics and that showed some variability in both polymorphism and divergence, according to gene annotations from GENCODE release 27 (22) and PopHuman polymorphism and divergence data (Figure 1). MKT-derived calculations were performed using the R package iMKT (https://github.com/BGD-UAB/iMKT; last accessed: February 2018). In total, eight different neutrality tests were performed. They identify different types of signatures that remain visible in the genomic sequences for a timescale ranging from few thousands of years after a single sweep selection event to several million years for the case of recurrent selection (4,23).

#### Linkage Disequilibrium (LD)

Selection signatures in the LD, e.g. long haplotypes, were detected using two complementary measures: *iHS* (24) and *XP-EHH* (9). *iHS* has good power to detect selective sweeps with haplotypes at moderate frequency (50–80%), while *XP-EHH* is more powerful for detecting selective sweeps when the selected haplotype has a frequency >80%. In the case of *XP-EHH*, which analyzes pairs of populations, only pairs CEU-YRI, CEU-CHB and YRI-CHB were considered, and the population showing the evidence of selection was identified with the locus-specific branch length method (*LSBL*) (25). These long-range haplotypes persist for relatively short periods of type, typically up to ∼30 kya, and thus this signature allows us to identify recent selection events only (4).

#### Site Frequency Spectrum (SFS)

Five statistics have been considered: four are based on both the allele frequency spectrum and the levels of variability—Fay and Wu’s *H* (26), Fu and Li’s *D* and *F* (27), and Tajima’s *D* (28)—, and the other one is based on population differentiation—*F_ST_* (29,30)—. Fay and Wu’s *H* detects an excess of high-frequency derived SNPs, compatible with an incomplete sweep or recombination breaking swept linked SNPs. Fu and Li’s *D* and *F* and Tajima's *D* assess the lack or excess of rare alleles (or singletons). Lack of rare alleles is compatible with balancing selection, while an excess is normally explained by either positive or weakly deleterious selection. *F_ST_* detects population-specific selective events that changed the genetic composition of the affected population. It analyzes pairs of populations. The pairs CEU-YRI, CEU-CHB and YRI-CHB were considered, and the population showing the evidence of selection was identified with the *LSBL* (25). SFS signatures can persist in the genomes for a longer period than LD, and thus selective events identified by shifts in the SFS might have occurred up to ∼80 kya (4).

#### Protein changes

Recurrent selection since the split between our species and chimpanzees (<6 mya) is detected using a test based on comparisons of polymorphism and divergence—*MKT* (19)—, and the result of the test is summarized with the estimator *α* (20,21). For this calculation, we used an MKT-based methodology that corrects for the presence of non-synonymous slightly deleterious segregating sites in order to avoid underestimating *α* (31).

### Genome-wide scan of selection

For the parameter *α* of MKT, evidence of positive selection for protein-coding genes was inferred when *α* > 0 and the Fisher’s Exact Test for the 2 × 2 MKT contingency table was significant (*P*-value < 0.05) (Figure 1). Because the other seven selection statistics have not been associated with a simple parametric distribution, candidate windows under selection were identified as the most extreme values (within the 0.05% tail) in the corresponding empirical distribution. These empirical distributions were performed independently for each of the 22 populations (or three population pairs in the case of *XP-EHH* and *F_ST_*), and independently for the autosomes and the X chromosome (to account for different demographic histories and the different effective population size of the autosomes compared to the X chromosome; chromosome Y was not analyzed). In total, 116 empirical distributions were obtained for autosomal regions, and 91 for the X chromosome (data of *iHS* and *XP-EHH* was not available for the X chromosome in PopHuman) (Figure 1).

From the initial 186 549 10-kb non-overlapping windows from PopHuman for each population and statistic, those containing <5 segregating sites were discarded (<0.2%) (Supplementary Figure S1). Then, an empirical *P*-value was assigned to each of the remaining windows for each of the 116 combinations of population (or population pair) and statistic, separately for the autosomes as a whole and the X chromosome. Specifically, for each window *i* in a population (or population pair), *p* is the quantile of that window for statistic *j*, that is, its empirical *P*-value. In the case of Tajima’s *D*, Fu and Li’s *D* and *F*, and Fay and Wu’s *H*, two-tailed *P*-values were calculated. Once the significance for each individual 10-kb window in the genome was assessed, a candidate region under selection was defined as being a contiguous genomic region containing at least one 10-kb significant window (*P*-value < 0.0005) and spanning adjacent windows with *P*-values < 0.005 (Supplementary Figure S2). In addition, this region may span stretches <20-kb of contiguous nucleotides not analyzed in PopHuman (i.e. because they contain non-accessible bases according to the Pilot-style Accessibility Mask of the 1000GP (13,18)). This outlier approach was designed to face the unique features and limitations of our PopHuman source data and to be highly conservative defining candidate regions under selection. We expect that it likely results in an enriched set of genomic regions that have been targets of natural selection along the human evolutionary history (11), and refer to the outlier regions as candidate regions showing signatures of selection.

Once candidate selected regions (or genes) were assigned for the 22 populations (or three population pairs) and eight statistics, they were collapsed according to their coordinates into a joint set of 2879 candidate regions under selection genome-wide. Of these, 20 regions were removed because they were completely located in DAC Blacklisted regions (i.e. regions of the reference genome which are troublesome for high throughput sequencing aligners) or partially overlapped genomic gaps, as obtained from the UCSC (32). Therefore, a total of 2859 regions were finally considered.

### Structural and functional annotations

The final 2859 candidate regions under selection were structurally and functionally characterized according to 15 different annotations categorized into five groups, extracted from the UCSC (32) and two publicly available databases (33,34) (Figure 1).

#### Sequencing

(i) *Mappability* was assessed as the percentage of bases in the region that do not present any troublesome to high-throughput sequencing aligners according to the DAC Blacklisted regions of the UCSC (35). (ii) *Distance to closest GAP* was computed as the distance (in Mb) to the closest gap (32).

#### Regulation

(iii) *CpG Islands* (36), (iv) *Vista Enhancers* (37), (v) *Transcription Factor Binding Sites* (TFBSs) (32) and (vi) *ORegAnno Regulatory Elements* (38) were computed as the total number of elements contained in the region, as well as the number of SNPs contained in the overlapping elements.

#### Comparative genomics

Evolutionary conservation of the regions was assessed by considering the results of three different algorithms—phastCons, PhyloP and GERP—on the multiple alignments of the genomes of 100 vertebrate species (39). (vii) *PhyloP Evolutionary Conservation* and (viii) *GERP Constrained Elements* were assessed as the percentage of bases in the region that have a score >2 for the given statistic (i.e. constrained sites) (32). (ix) *phastCons Evolutionary Conservation* was calculated as the percentage of bases that overlap phastCons conserved elements (32).

#### Structural variation

(x) *InvFEST Inversions* (34), (xi) *DGV Structural Variants* (40), (xii) *RepeatMasker* (41), (xiii) *Segmental Duplications* (42) and (xiv) *TRF Simple Tandem Repeats* (43) were assessed as the percentage of bases in the region that overlap these genomic elements.

#### Archaic introgression

(xv) *Archaic introgression* was assessed as the percentage of bases in the region that overlap either Neanderthal or Denisova introgressed haplotypes (33).

### Published references

A total of 268 publications from 1954 to 2018 reporting either specific loci or multiple regions from a genome-wide scan of selection in the human genome were cross-referenced with our final 2859 candidate regions under selection (Figure 1 and Supplementary Table S2). Of these, 132 publications were directly extracted from the dbPSHP database (15), while the other 136 were manually curated here. Exhaustive information from the main text and/or supplementary figures and tables was extracted for each reported loci, including the genomic coordinates, affected population(s), statistic(s), type of selection and PubMed ID. Genomic coordinates were lifted over to GRCh37/hg19 using the LiftOver tool of the UCSC (32), or deduced from protein-coding gene location, if necessary.

## OVERVIEW OF THE POPHUMANSCAN ONLINE CATALOG

**Figure 2.**
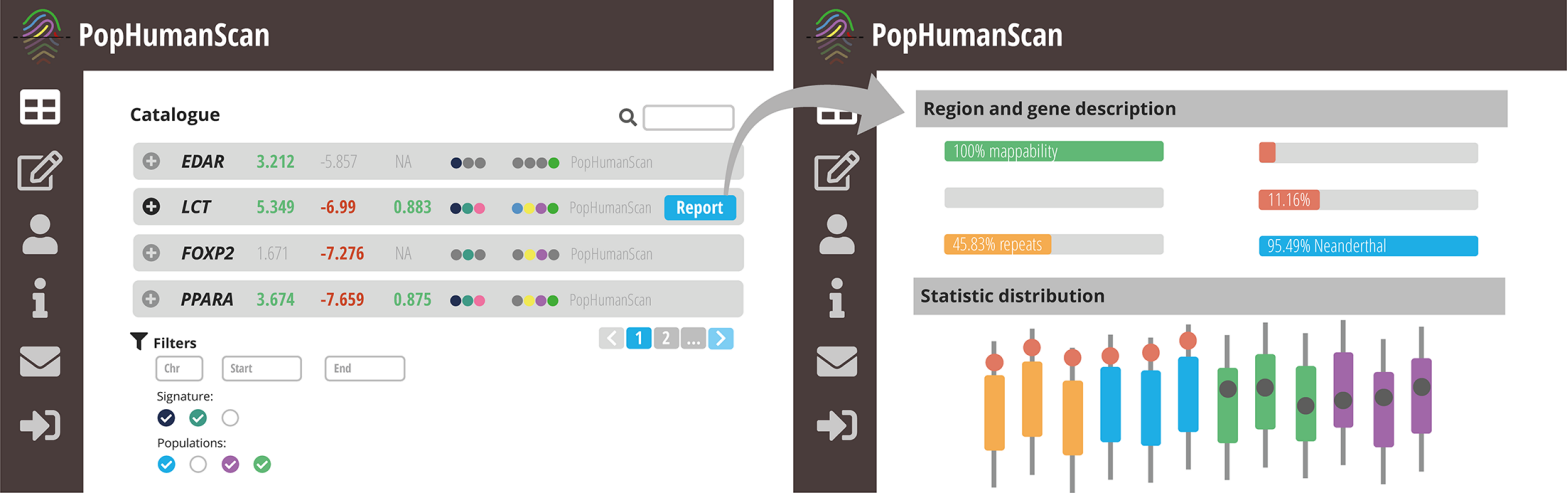
Simplified representation of the PopHumanScan interface. The main PopHumanScan table is displayed to the left, while the complete report for a particular candidate region under selection is displayed to the right.

In addition to the exhaustive genome-wide selection scan that has been performed, we have also created PopHumanScan, a collaborative, online database that is aimed at compiling and annotating adaptation events along the human evolutionary history (Figure 2). PopHumanScan reports each evidence of selection with the empirical distributions of the corresponding DNA diversity statistic across the human genome and among populations, structural and functional annotations of the region, links to external databases, as well as cross-references to 268 publications.

### Implementation

PopHumanScan is currently running under Apache on a CentOS 7.2 Linux x64 server with 16 Intel Xeon 2.4 GHz processors and 32 GB RAM. It is mainly built on PHP as backend framework. It also includes AJAX for specific file requests and MySQL for data storage. The client-side is build on JavaScript and uses several JavaScript libraries, including jQuery, the jQuery plugin DataTables and Plotly.js, as well as a custom Bootstrap 4 framework.

### The PopHumanScan catalog

#### Main table

All 2859 candidate regions under selection are displayed as rows in an interactive table (Figure 2, left). The information displayed in each row includes: (i) the genomic coordinates of the candidate locus, (ii) genes contained in or partially overlapping the region (if any), (iii) the most extreme value for each of the eight statistics considered (i.e. most extreme value in any 10-kb window included in the region, for any population or population pair; green (positive) and red (negative) values are outliers (*P* < 0.0005) in the corresponding empirical distribution), (iv) color-coded dots depicting different types of selection signatures (i.e. 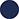
*LD*, 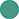
*SFS* and/or 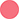
*Protein Changes*), (v) color-coded dots depicting the meta-population(s) that show signatures of selection (i.e. 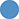
*Europe* (*EUR*), 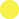
*Africa* (*AFR*), 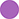
*South-Asia* (*SAS*) and/or 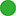
*East-Asia* (*EAS*)) and (vi) the source that contributed the candidate region under selection. At the time of writing, all 2859 regions came uniquely from our genome-wide selection scan (i.e. source labeled as *PopHumanScan*), but additional data sources by contributors from the scientific community are expected once PopHumanScan is published (see next section). By clicking the ⊕ icon at the beginning of each row, detailed information of the particular candidate region under selection is displayed, including the values for all significant statistics in all target populations (or population pairs) and an overview of the main structural and functional annotations and cross-referenced publications (i.e. non-gray buttons represent overlapping annotations or cross-referenced publications), as well as access to the complete report for the corresponding candidate region under selection. At last, several filters are available at the bottom of the page to narrow the search.

#### Complete report

A complete report for each candidate region can be accessed from the main table (Figure 2, right). The first section of the report displays all the structural and functional annotations of the region, together with links to external databases: (i) *PopHuman* (18), which complements the population genomics information; (ii) *HaploReg* (44), which allows the exploration of evolutionary conservation, expression eQTSs, epigenomic data and regulatory annotations; and (iii) *Ensembl* (45), which allows the exploration of the LD of the region, among others. The second section lists all the genes contained in or partially overlapping the region (if any). For each encoded gene, a short description of the gene and associated *Gene Ontology* terms for the *Biological Process* classification (46) are provided, along with links to external databases: *Ensembl* (45), *NCBI* (47), *Uniprot* (48), *UCSC* (32), *Expression Atlas* (49), *OMIM* (50), *Open Targets* (51) and *HumanMine* (52). The third section contains cross-referenced publications that support the selection evidence found in the region. The fourth section contains an interactive graph showing recombination rate values in cM/Mb along the chromosome in which the region is located, calculated from the recombination map by Bhérer *et al.* (53) and extracted from PopHuman (18). The specific location of the candidate region under selection is indicated with dashed vertical lines, and the solid horizontal line represents the average recombination rate value in the candidate region. At last, in the fifth section boxplots show the distribution of each significant statistic in all the populations (or population pairs). Highlighted values correspond to those in the candidate region, and those in red are outliers of the empirical distribution (*P* < 0.0005).

### Utilities and support resources

#### Contributing to PopHumanScan

PopHumanScan has been devised as a collaborative database. In order to incorporate information contributed by the scientific community, two password-protected tools have been implemented. The first one allows users to add additional candidate regions under selection in the catalog. All contributed regions will be subjected to manual curation and clearly labeled with a data source tag. The second tool allows manually cross-referencing candidate regions already present in the database.

#### Help and tutorial

This section documents the data used and the procedures implemented in PopHumanScan, as well as instructions on how to contribute to it. Interestingly, it also contains a complete tutorial introducing to the usage of the database through a step-by-step worked example.

## CONTENTS OF POPHUMANSCAN

**Figure 3.**
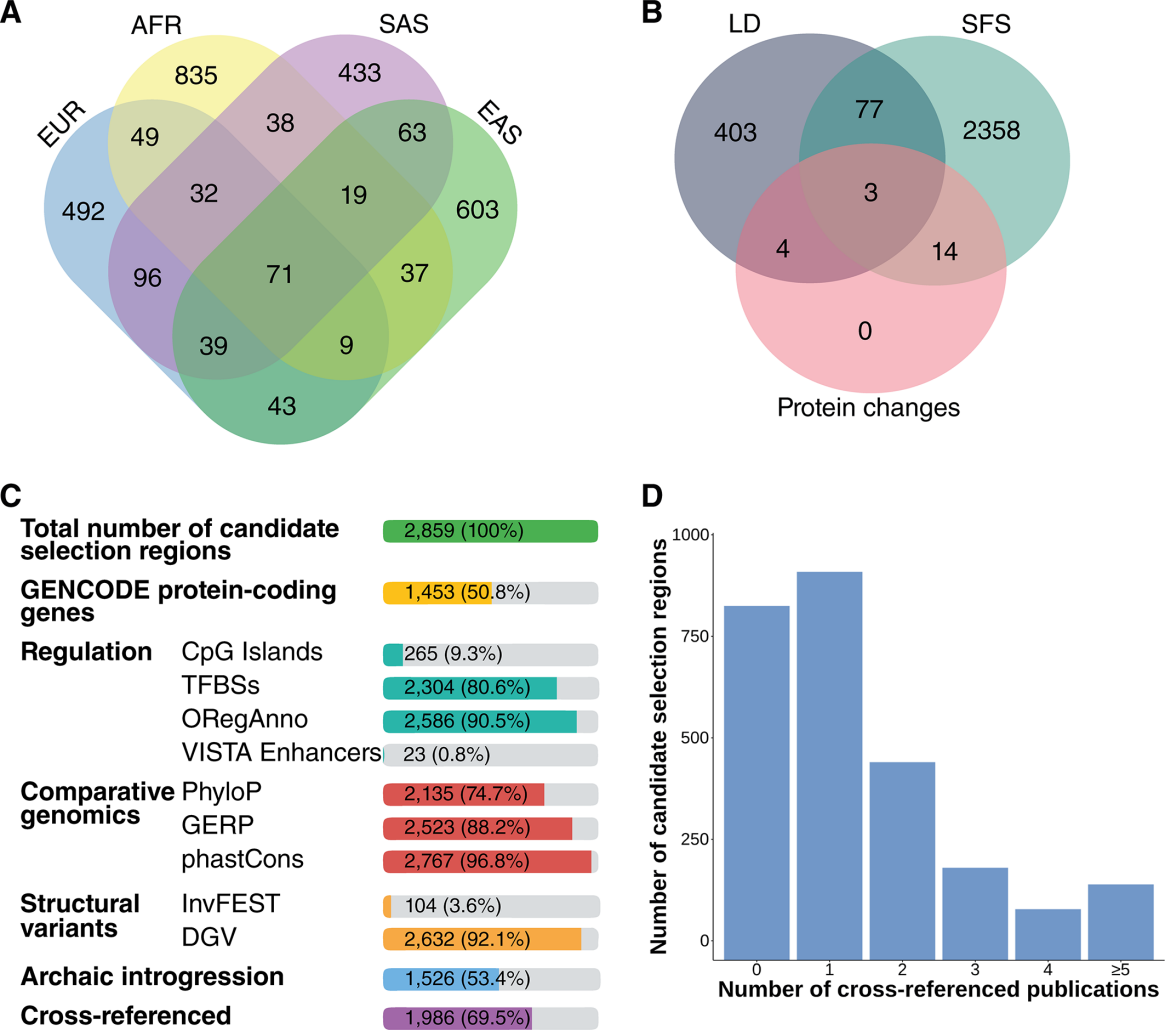
Summary of the contents of PopHumanScan. (**A**) Number of candidate regions under selection unique and shared among the four meta-populations: 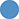
*Europe (EUR)*, 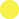
*Africa (AFR)*, 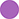
*Sourth-Asia (SAS)* and 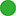
*East-Asia (EAS)*. (**B**) Number of candidate regions under selection unique and shared among the three different signature types: 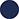
*Linkage Disequilibrium (LD)*, 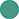
*Site Frequency Spectrum (SFS)* and 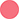
*Protein Changes*. (**C**) Number of candidate regions under selection overlapping different structural and functional annotations. (**D**) Number of candidate regions under selection cross-referenced with 0, 1, 2, 3, 4 or ≥5 published papers.

At the time of writing, the PopHumanScan database contains 2859 candidate regions under selection derived from the genome-wide selection scan pipeline presented here. Regions are distributed homogeneously along the autosomes and the X chromosome (Table 1 and Supplementary Figure S3). Of these, 1453 regions (50.8%) overlap GENCODE protein-coding genes, and 1986 regions (69.5%) are cross-referenced with at least one publication (Table 1 and Figure 3).

**Table 1.** Summary of the candidate regions under selection included in PopHumanScan

		Regions with selection signatures in meta-populations				Regions with different types of signatures				
Chromosome	Number of candidate regions	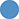 *European* (EUR)	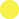 *African* (AFR)	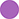 *South-Asian* (SAS)	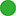 *East-Asian* (EAS)	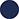 *Linkage disequilibrium (LD)*	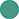 *Site Frequency Spectrum (SFS)*	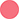 *Protein Changes*	Regions overlapping protein-coding genes	Regions cross-referenced with publications
1	214	57	84	48	66	46	176	2	123	152
2	253	77	95	60	81	56	203	2	131	191
3	201	61	81	49	54	34	173	0	116	153
4	241	62	110	68	59	42	207	1	111	173
5	166	46	62	41	52	32	140	1	85	119
6	171	42	83	42	49	29	146	1	81	118
7	144	53	58	49	49	25	125	0	74	115
8	164	41	57	51	48	32	135	0	68	108
9	96	28	33	22	34	12	85	1	51	59
10	141	45	47	45	48	21	126	1	70	103
11	120	35	37	34	44	22	99	2	62	86
12	142	44	52	35	42	24	123	1	87	105
13	82	17	31	27	21	9	75	0	23	51
14	74	19	29	25	22	15	61	0	39	57
15	79	34	29	17	25	12	70	1	47	57
16	88	20	20	41	30	21	68	1	49	69
17	86	30	32	34	24	18	71	1	64	69
18	56	15	23	14	18	7	49	0	24	43
19	67	21	29	21	27	5	62	4	42	38
20	59	19	22	14	23	10	52	1	29	49
21	34	13	11	10	16	7	28	0	11	26
22	39	8	16	8	15	8	35	1	27	32
X	142	44	49	36	37	ND	142	0	39	13
**TOTAL**	**2859**	**831**	**1090**	**791**	**884**	**487**	**2451**	**21**	**1453**	**1986**
		***29.1%***	***38.1%***	***27.7%***	***30.9%***	***17.0%***	***85.7%***	***0.7%***	***50.8%***	***69.4%***

ND = Not Determined

### Selection signatures in meta-populations

The total number of candidate regions showing signatures of selection in the four meta-populations is: 831 (29.1%) in EUR, of which 413 (49.7%) overlap protein-coding genes; 1090 (38.1%) in AFR, of which 580 (53.2%) overlap protein-coding genes; 791 (27.7%) in SAS, of which 401 (50.7%) overlap protein-coding genes; and 884 (30.9%) in EAS, of which 424 (48.0%) overlap protein-coding genes (Table 1). Most of the regions (82.5%) show signatures that are unique to one single meta-population (Figure 3A): 492 (17.2%) show signatures that are unique in EUR, 835 (29.2%) are unique in AFR, 433 (15.1%) are unique in SAS and 603 (21.1%) are unique in EAS. Of the 1090 regions showing signatures in AFR, 76.6% are unique to AFR; while a lesser percentage—59.2, 54.7 and 68.2%—of the regions showing signatures in EUR, SAS and EAS, respectively, are unique to their meta-population. About one third (29.0%) of the candidate regions under selection are shared across populations within the same meta-population. This percentage is higher for candidate regions showing both LD and SFS signatures (52.7%), it is 33.6% for candidate regions showing LD signatures only and 27.1% for candidate regions showing SFS signatures only.

### Types of selection signature

The total number of candidate regions showing distinct types of signatures of selection is: 487 (17.0%) for *LD*; 2451 (85.7%) for *SFS*; and 21 (0.7%) for *Protein Changes* (i.e. recurrent selection since the split between humans and chimpanzees) (Table 1). Most of the regions (96.6%) show one single signature of selection (Figure 3B): 403 (14.1%) show LD signatures only; and 2358 (82.5%) show SFS signatures only. All genes showing evidence of recurrent selection also show signatures in either LD and/or SFS, as only genes overlapping candidate regions under selection detected by LD and/or SFS were tested for *α* (MKT). These results would indicate that the statistics we used in our genome-wide scan of selection look at different characteristics of the genetic variability of the region, and that they are largely complementary.

### Structural description of the regions

#### Region length

Most of the candidate regions under selection (63.6%) span one single 10-kb window, and the variable *lengths of candidate regions* follows a reversed J-shaped distribution (Supplementary Figure S4A).

#### Distance between consecutive regions

The average distance between consecutive candidate regions is ∼1 Mb, and the distribution of distances is also reversed J-shaped (Supplementary Figure S4B).

#### Recombination

The average recombination rate of the candidate regions is 0.71 cM/Mb, and the distribution of recombination rates is again reversed J-shaped (Supplementary Figure S4C). There is a strong, negative, non-linear association between recombination rate and both region length (Supplementary Figure S5A) and distance between consecutive candidate regions (Supplementary Figure S5B).

### Functional description of the regions

#### Regulation

Most of the candidate regions (90.5%) contain at least one regulatory element annotated in the ORegAnno database, and 80.6% contain TFBSs (Figure 3C). On the contrary, VISTA enhancers are much less abundant in the genome and they are only found in 23 of the 2859 candidate regions (0.8%). CpG Islands are also in shortage and they are present in 9.3% of the regions.

#### Comparative genomics

Nearly all (96.8%) candidate regions overlap phastCons conserved elements. In the case of GERP and PhyloP, 88.2 and 74.7% of the regions, respectively, overlap constrained bases with score >2.

#### Structural variation

The Database of Genomic Variants (DGV) (40) is a very exhaustive database of structural variants annotated in the human genome. One or more elements annotated in this database are present in 92.1% of the candidate regions under selection. On the contrary, only 104 regions (3.6%) overlap validated polymorphic inversions from the manually curated InvFEST database (34).

#### Archaic introgression

A total of 1526 of the candidate regions (53.4%) overlap haplotypes introgressed from either neanderthals or denisovans. This percentage is expected, as introgressed haplotypes persisting in different present-day human individuals cover 46.7% of the reference genome (33).

#### Cross-references with publications

A percentage of 69.5% of the candidate regions are cross-referenced with at least one publication, and 36.0% are cross-referenced more than once (Figure 3D).

### Gene ontology analysis

Our candidate regions overlap a total of 1447 unique GENCODE protein-coding genes. These were functionally classified into Gene Ontology (GO) terms (46) according to the PANTHER GO-Slim annotation dataset using the PANTHER Classification System (54) (Supplementary Figure S6). In addition, statistically over- and under-represented functions were analyzed using the complete GO annotation dataset (46) using the same tool (Supplementary Tables S3–5). Interestingly, among all Biological Process categories, *regulation of neuron projection development* is over-represented (fold enrichment 1.88, False Discovery Rate (FDR) 1.23E-02), in addition to *cellular component organization* (fold enrichment 1.24, FDR 1.59E-03) (Supplementary Table S4). Finally, several Cellular Component categories are statistically over-represented, including *presynaptic membrane* (fold enrichment 2.72, FDR 4.32E-02) (Supplementary Table S5). In spite of finding some statistically over-represented GO categories in our genes list, selection signatures seem to be heterogeneous and a detailed analysis of each candidate region is required to understand the real story under each selective event.

## POPHUMANSCAN WITH AN EXAMPLE: SELECTION AT THE LACTASE LOCUS

The introduction of agriculture and cattle domestication in the Middle East and North Africa ∼10 000 years ago lead to strong selection pressure for the ability to digest milk as adults. This is accomplished if the enzyme lactase that metabolizes lactose, encoded by the *LCT* gene, maintains high levels into adulthood, a characteristic that is called lactase persistence. Several variants near the *LCT* locus show some of the strongest signals of selection in the human genome for those populations that have traditionally practiced dairying, including a genetic variant in an intron of the gene *MCM6*, upstream of *LCT* (3).

**Figure 4.**
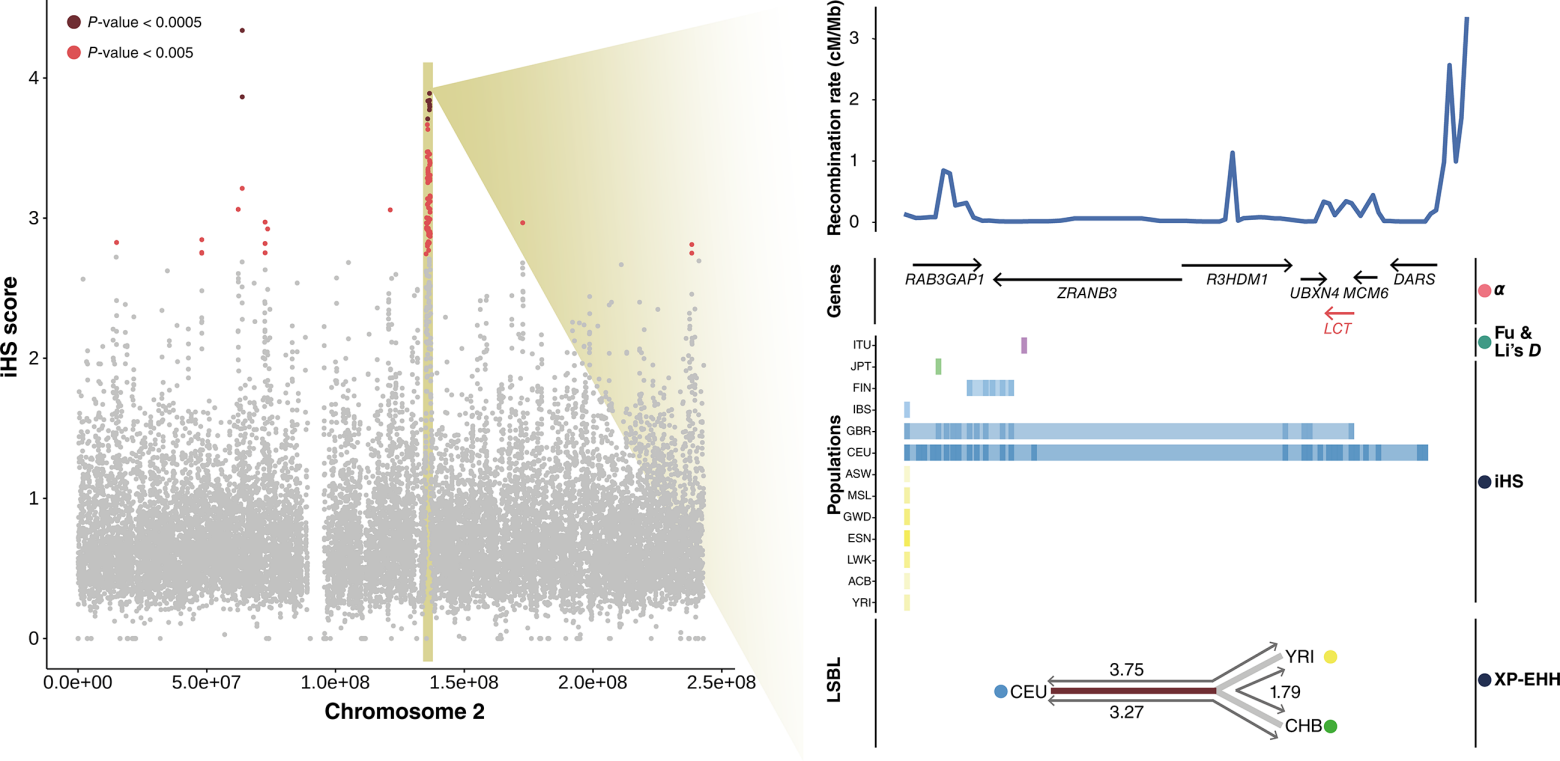
Signatures of selection detected at the lactase locus. The distribution of *iHS* values for the CEU population in 10-kb windows along chromosome 2 are displayed to the left; windows with a *P*-value < 0.0005 or *P*-value < 0.005 in the empirical distribution are highlighted. The candidate region under selection including the *LCT* gene is zoomed-in to the right, where all significant signatures at four different statistics spanning three different signature types are represented: 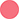
*Protein Changes*, 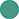
*Site Frequency Spectrum (SFS)* and 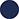
*Linkage Disequilibrium (LD)*. Signatures in each population are colored according to its meta-population: 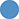
*Europe (EUR)*, 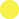
*Africa (AFR)*, 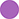
*South-Asia (SAS)* and 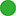
*East-Asia (EAS)*.

The *LCT* locus is found inside the longest candidate region under selection reported in PopHumanScan (∼1 Mb). The region is located in the long arm of chromosome 2 and contains 8 GENCODE protein-coding genes, including *LCT* and *MCM6* (Figure 4). Our genome-wide scan of selection has detected signatures at four different statistics that span the three types of signatures: LD (*iHS* and *XP-EHH*), SFS (Fu and Li's *D*) and Protein Changes (*α*). LD signatures involve basically EUR and AFR populations, while the signature of recurrent selection is more general to the four meta-populations. The region contains thousands of TFBSs and hundreds of ORegAnno regulatory elements, it overlaps evolutionary constrained elements, and >95% of the region overlaps haplotypes introgressed from Neanderthals. It has been reported in 24 published articles (of the set of 268 that we considered).

## CONCLUSION

In summary, our exhaustive approach combining eight different statistics to detect candidate regions under selection in 22 non-admixed human populations has been able to locate distinct signatures in 2859 regions that stand out from the background genomic variability, including abnormally long haplotypes, shifts in the SFS or excess of non-synonymous substitutions between our species and chimpanzees. Many of these regions probably manifest the footprints of selective sweeps that occurred at different historical ages, or recurrent selection that has been taking place during the last millions of years. The PopHumanScan online database is going to facilitate the thorough analysis of candidate regions under selection in the human genome by putting together all these evidences of selection with structural and functional annotations of the regions and cross-references to previously published articles. Furthermore, the database can incorporate new data from the scientific community through specific build-in utilities. All in all, PopHumanScan aims to become a central repository to share information, guide future studies and contribute to the research on human genome adaptation.

## DATA AVAILABILITY

Scripts for the PopHumanScan analysis pipeline are available as Jupyter Notebooks at https://github.com/BGD-UAB/PopHumanScan. All data, tools and support resources provided by the PopHumanScan database are freely available at https://pophumanscan.uab.cat. Log-in information to contribute data to PopHumanScan is available upon request.

## Supplementary Material

Supplementary Data
