# Supplementary material for: PopHumanScan: the online catalog of human genome adaptation
Source: Nucleic Acids Res. 2018 Oct 18;47(Database issue):D1080–9. doi: 10.1093/nar/gky959 (PMC6323894; doi:10.1093/nar/gky959)
Supplement: Supplementary Data [file gky959_supplemental_files.zip › PopHumanScan NAR2018 SupplementaryData Revised.pdf]

PopHumanScan: the online catalog of human genome adaptation

Jesús Murga-Moreno, Marta Coronado-Zamora, Alejandra Bodelón, Antonio Barbadilla* and Sònia Casillas*

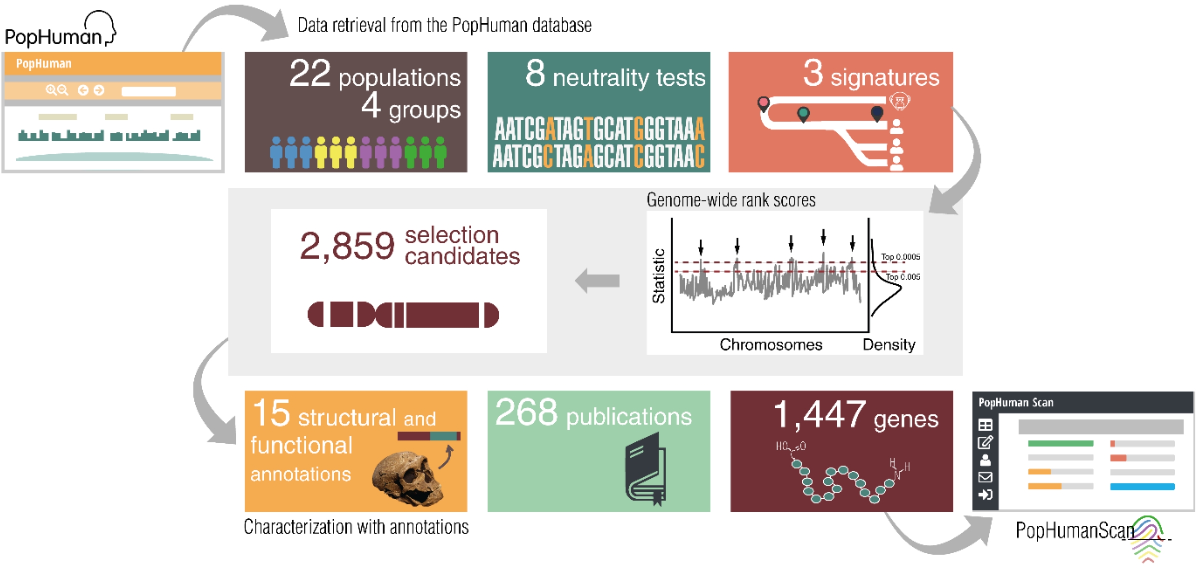

SUPPLEMENTARY DATA

Table S1. List of 22 non-admixed populations analyzed in PopHumanScan, derived from the 1000GP Phase III.

Population Code	Population Description	Metapopulation	Sample Size
CDX	Chinese Dai in Xishuangbanna, China	*East-Asian* (EAS)	93
CHB	Han Chinese in Beijing, China	*East-Asian* (EAS)	103
CHS	Southern Han Chinese	*East-Asian* (EAS)	105
JPT	Japanese in Tokyo, Japan	*East-Asian* (EAS)	104
KHV	Kinh in Ho Chi Minh City, Vietnam	*East-Asian* (EAS)	99
CEU	Utah residents (CEPH) with Northern and Western European ancestry	*European* (EUR)	99
GBR	British in England and Scotland	*European* (EUR)	91
FIN	Finnish in Finland	*European* (EUR)	99
IBS	Iberian Populations in Spain	*European* (EUR)	107
TSI	Toscani in Italia	*European* (EUR)	107
ESN	Esan in Nigeria	*African* (AFR)	99
GWD	Gambian in Western Division, Mandinka	*African* (AFR)	113
LWK	Luhya in Webuye, Kenya	*African* (AFR)	99
MSL	Mende in Sierra Leone	*African* (AFR)	85
YRI	Yoruba in Ibadan, Nigeria	*African* (AFR)	108
ACB	African Caribbean in Barbados	*African* (AFR)	96
ASW	People with African Ancestry in Southwest USA	*African* (AFR)	61
BEB	Bengali in Bangladesh	*South-Asian* (SAS)	86
GIH	Gujarati Indians in Houston, TX, USA	*South-Asian* (SAS)	103
ITU	Indian Telugu in the UK	*South-Asian* (SAS)	102
PJL	Punjabi in Lahore, Pakistan	*South-Asian* (SAS)	96
STU	Sri Lankan Tamil in the UK	*South-Asian* (SAS)	102

Table S2. Compendium of candidate regions under selection extracted from 268 publications. (Table in XLS format)

Table S3. Statistical over-representation test of Gene Ontology terms in 1,447 GENCODE protein-coding genes overlapping our candidate regions under selection, according to the GO Molecular Function classification (released 2018/07/03) and the PANTHER over-representation test (released 2017/12/05). Significance was tested with Fisher's Exact Test with FDR multiple test correction.

GO Molecular Function	Homo sapiens - REFLIST (21042)	PopHumanScan (1476)	PopHumanScan (expected)	Over/Under	Fold enrichment	P-value	FDR
adenyl ribonucleotide binding (GO:0032559)	1557	154	109.22	+	1.41	4.59E-05	3.56E-02
↳ adenyl nucleotide binding (GO:0030554)	1569	154	110.06	+	1.4	7.42E-05	4.93E-02
↳ binding (GO:0005488)	15105	1163	1059.55	+	1.1	2.94E-09	6.84E-06
ion binding (GO:0043167)	6239	513	437.64	+	1.17	4.37E-05	4.06E-02
protein binding (GO:0005515)	11830	947	829.82	+	1.14	2.13E-09	9.90E-06

Table S4. Statistical over-representation test of Gene Ontology terms in 1,447 GENCODE protein-coding genes overlapping our candidate regions under selection, according to the GO Biological Process classification (released 2018/07/03) and the PANTHER over-representation test (released 2017/12/05). Significance was tested with Fisher's Exact Test with FDR multiple test correction.

GO Biological Process	Homo sapiens - REFLIST (21042)	PopHumanScan (1476)	PopHumanScan (expected)	Over/Under	Fold enrichment	P-value	FDR
regulation of neuron projection development (GO:0010975)	477	63	33.46	+	1.88	8.66E-06	1.23E-02
↳ regulation of biological process (GO:0050789)	11443	885	802.67	+	1.1	3.07E-05	3.70E-02
↳ biological regulation (GO:0065007)	12104	950	849.04	+	1.12	2.45E-07	1.28E-03
↳ regulation of cellular process (GO:0050794)	10758	848	754.62	+	1.12	2.70E-06	6.05E-03
↳ regulation of multicellular organismal process (GO:0051239)	2904	262	203.7	+	1.29	3.96E-05	4.42E-02
↳ nervous system development (GO:0007399)	2245	217	157.48	+	1.38	3.74E-06	7.32E-03
↳ anatomical structure development (GO:0048856)	5179	443	363.28	+	1.22	5.45E-06	9.48E-03

↳ developmental process (GO:0032502)	5501	466	385.87	+	1.21	7.14E-06	1.12E-02
↳ multicellular organismal process (GO:0032501)	6697	550	469.76	+	1.17	1.97E-05	2.57E-02
↳ cellular process (GO:0009987)	15086	1147	1058.21	+	1.08	4.42E-07	1.73E-03
cellular component organization (GO:0016043)	5448	472	382.15	+	1.24	5.07E-07	1.59E-03
↳ cellular component organization or biogenesis (GO:0071840)	5622	480	394.36	+	1.22	1.92E-06	5.01E-03

Table S5. Statistical over-representation test of Gene Ontology terms in 1,447 GENCODE protein-coding genes overlapping our candidate regions under selection, according to the GO Cellular Component classification (released 2018/07/03) and the PANTHER over-representation test (released 2017/12/05). Significance was tested with Fisher's Exact Test with FDR multiple test correction.

GO Cellular Component	Homo sapiens - REFLIST (21042)	PopHumanScan (1476)	PopHumanScan (expected)	Over/Under	Fold enrichment	P-value	FDR
presynaptic membrane (GO:0042734)	84	16	5.89	+	2.72	7.67E-04	4.34E-02
↳ neuron part (GO:0097458)	1601	162	112.3	+	1.44	9.60E-06	1.19E-03
↳ cell part (GO:0044464)	17000	1284	1192.47	+	1.08	9.63E-10	9.54E-07
↳ cell (GO:0005623)	17027	1287	1194.37	+	1.08	5.53E-10	1.10E-06
↳ synapse part (GO:0044456)	752	84	52.75	+	1.59	8.47E-05	6.99E-03
↳ synapse (GO:0045202)	901	111	63.2	+	1.76	7.78E-08	3.08E-05
↳ synaptic membrane (GO:0097060)	328	43	23.01	+	1.87	2.79E-04	1.90E-02
↳ plasma membrane region (GO:0098590)	1098	115	77.02	+	1.49	6.03E-05	5.43E-03

↳ membrane (GO:0016020)	9701	782	680.48	+	1.15	3.28E-07	9.28E-05
↳ plasma membrane part (GO:0044459)	2850	269	199.91	+	1.35	1.27E-06	2.79E-04
↳ plasma membrane (GO:0005886)	5571	460	390.78	+	1.18	1.11E-04	8.47E-03
↳ cell periphery (GO:0071944)	5689	473	399.06	+	1.19	3.95E-05	3.72E-03
↳ membrane part (GO:0044425)	6926	573	485.83	+	1.18	4.16E-06	6.34E-04
transport vesicle membrane (GO:0030658)	193	28	13.54	+	2.07	8.50E-04	4.67E-02
↳ organelle part (GO:0044422)	9416	739	660.49	+	1.12	7.73E-05	6.66E-03
↳ organelle (GO:0043226)	13417	1023	941.14	+	1.09	1.52E-05	1.77E-03
↳ intracellular organelle (GO:0043229)	12628	968	885.8	+	1.09	2.21E-05	2.30E-03
↳ intracellular part (GO:0044424)	14425	1099	1011.85	+	1.09	1.55E-06	3.06E-04
↳ intracellular (GO:0005622)	14699	1123	1031.07	+	1.09	2.86E-07	9.44E-05
↳ cytoplasm (GO:0005737)	11502	889	806.81	+	1.1	3.05E-05	3.02E-03

↳ intracellular organelle part (GO:0044446)	9178	721	643.79	+	1.12	1.04E-04	8.22E-03
cytoplasmic region (GO:0099568)	483	56	33.88	+	1.65	7.47E-04	4.35E-02
cell junction (GO:0030054)	1271	133	89.15	+	1.49	1.66E-05	1.83E-03
nucleoplasm (GO:0005654)	3487	320	244.6	+	1.31	9.81E-07	2.43E-04
↳ nuclear lumen (GO:0031981)	4093	362	287.11	+	1.26	4.15E-06	6.85E-04
↳ intracellular organelle lumen (GO:0070013)	5219	424	366.09	+	1.16	8.84E-04	4.60E-02
↳ organelle lumen (GO:0043233)	5219	424	366.09	+	1.16	8.84E-04	4.73E-02
↳ membrane-enclosed lumen (GO:0031974)	5219	424	366.09	+	1.16	8.84E-04	4.49E-02
↳ nuclear part (GO:0044428)	4489	393	314.88	+	1.25	3.37E-06	6.07E-04
cytoskeleton (GO:0005856)	2171	199	152.29	+	1.31	2.22E-04	1.57E-02
↳ intracellular non-membrane-bounded organelle (GO:0043232)	4195	367	294.26	+	1.25	8.22E-06	1.16E-03

↳ non-membrane-bounded organelle (GO:0043228)	4195	367	294.26	+	1.25	8.22E-06	1.08E-03
plasma membrane bounded cell projection (GO:0120025)	2067	188	144.99	+	1.3	4.61E-04	2.85E-02
↳ cell projection (GO:0042995)	2135	195	149.76	+	1.3	2.84E-04	1.88E-02
protein-containing complex (GO:0032991)	5377	445	377.17	+	1.18	1.20E-04	8.81E-03
integral component of membrane (GO:0016021)	5467	446	383.49	+	1.16	4.30E-04	2.75E-02
↳ intrinsic component of membrane (GO:0031224)	5618	456	394.08	+	1.16	5.40E-04	3.24E-02

A

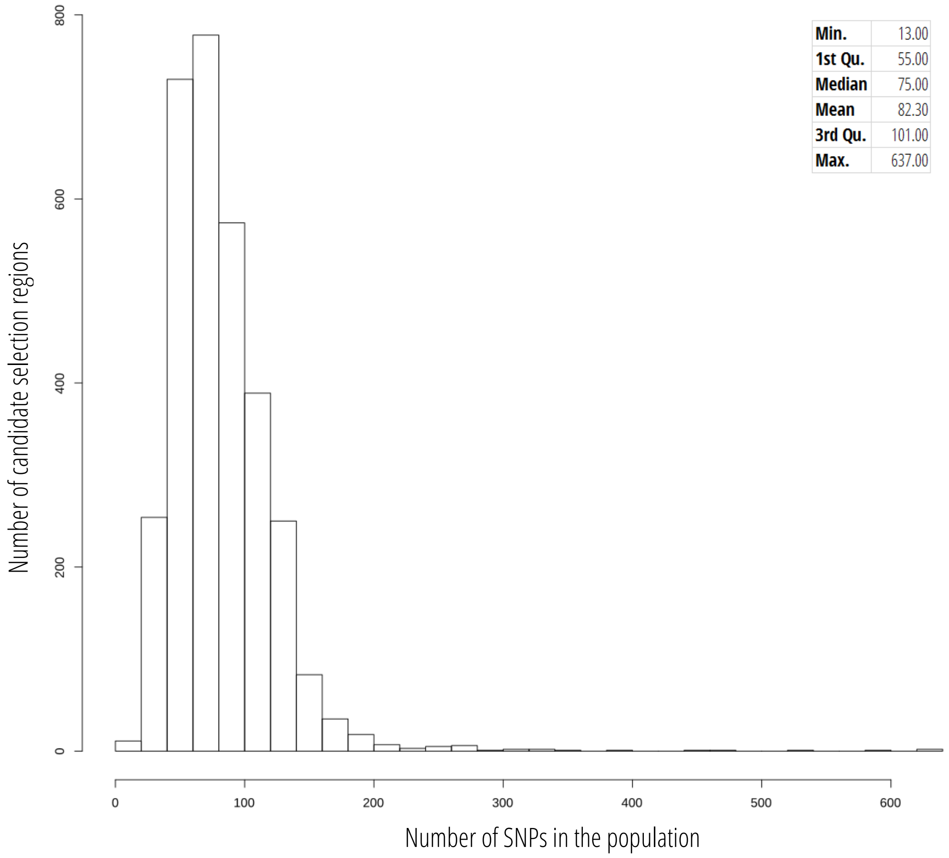

B

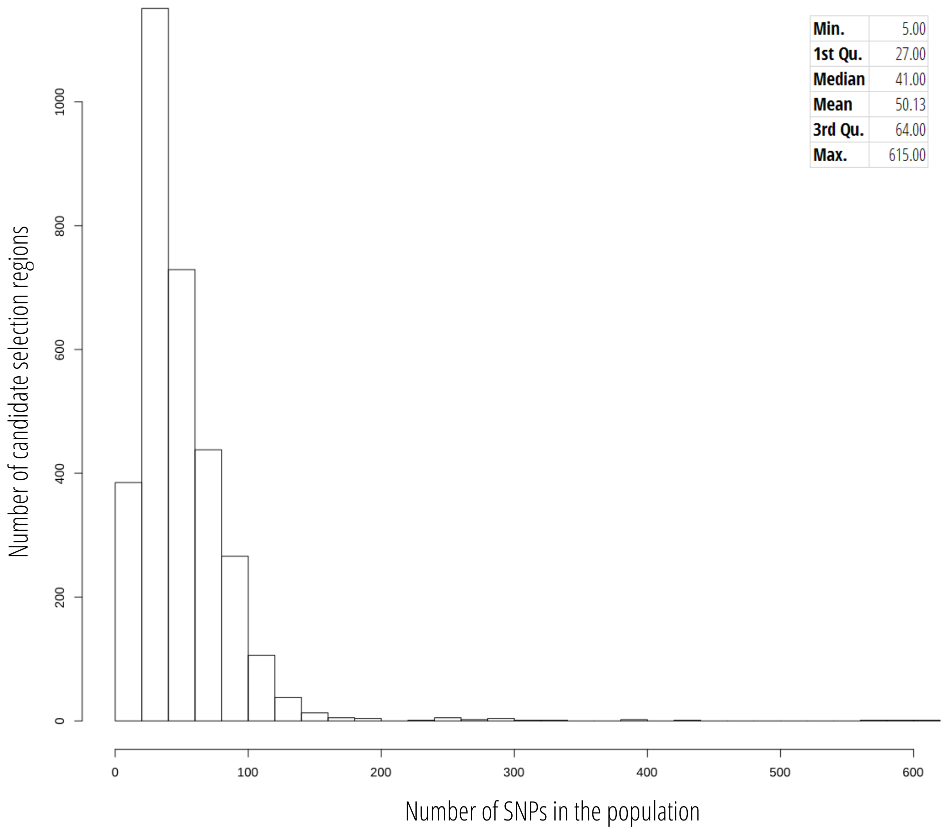

Figure S1. *(continues in the next page)*

C

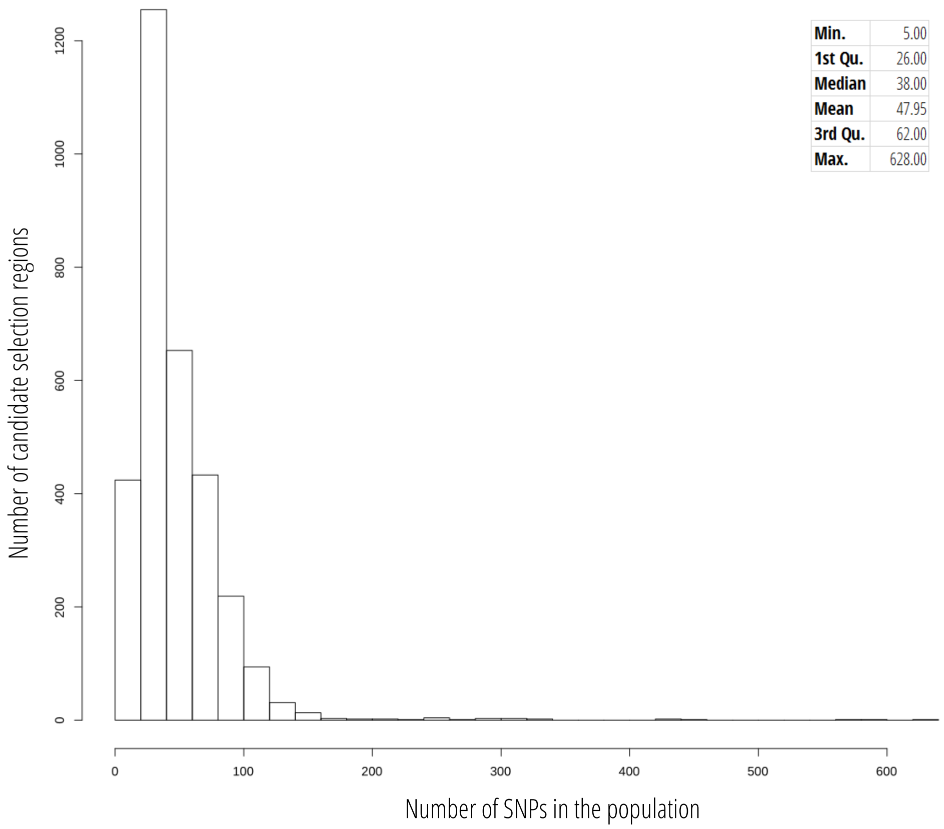

D

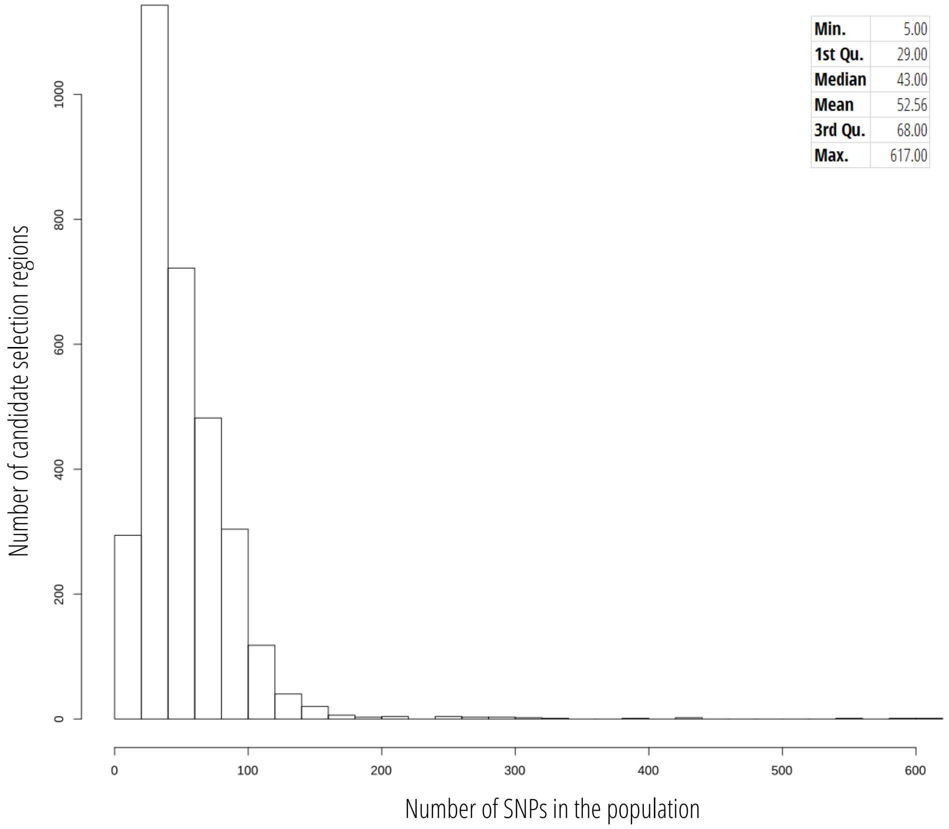

Figure S1. Distribution of the number of SNPs in the 10-kb analyzed windows for four representative populations, one of each human meta-population: (A) YRI (*African*), (B) CEU (*European*), (C) CHB (*East-Asian*), and (D) GIH (*South-Asian*).

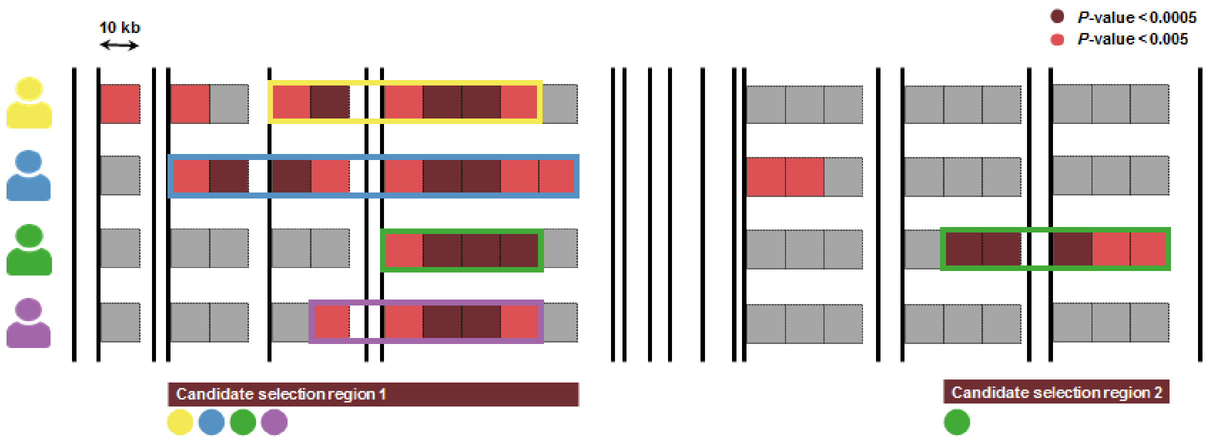

Figure S2. Definition of candidate regions under selection. Rows represent the results of one single variation statistic calculated along the region on a population. In this example, each of the four populations represented corresponds to a different human meta-population: ● *African*, ● *European*, ● *East-Asian*, and ● *South-Asian*. Squares represent 10-kb windows analyzed along this genomic region with PopHuman, while empty regions between squares represent regions that were not analyzed because they contain non-accessible bases (black vertical lines) according to the Pilot-style Accessibility Mask of the 1000GP [see Casillas *et al.* (2008) for details]. The color of the squares represents the *P*-value of the empirical distribution for the corresponding variation statistic and population: ● *P*-value < 0.0005, ● 0.0005 < *P*-value < 0.005, and ● *P*-value > 0.005. Rectangles spanning consecutive 10-kb windows along a row represent candidate regions under selection for the corresponding variation statistic and population, *i.e.*, contiguous genomic regions containing at least one 10-kb significant window (*P*-value < 0.0005) and spanning adjacent windows with *P*-value < 0.005. In addition, they may span stretches <20 kb of contiguous nucleotides not analyzed in PopHuman. In the case of the African population, the candidate region under selection does not extend to the windows with *P*-value < 0.005 to the left because there is a 10-kb window in the middle with *P*-value > 0.005. In the case of the European population, the candidate region under selection does not extend to the windows with *P*-value < 0.005 to the right because there is a stretch >20 kb of contiguous nucleotides not analyzed in PopHuman in the middle. On the contrary, regions within candidate regions under selection not analyzed in PopHuman are <20-kb long. Finally, candidate regions detected in each population are stacked to a final set of candidate regions under selection (maroon boxes at the bottom of the figure). In this example, two different candidate regions are detected: the first one with signals in the four meta-populations, and the second one with signals in the East-Asian meta-population. In the PopHumanScan analysis, empirical distributions are calculated for 7 different variation statistics and 22 populations (or 3 population pairs, depending on the variation statistic), so 116 empirical distributions are stacked simultaneously for autosomal regions (see text for details). Cited reference: *Casillas et al. (2018) Nucleic Acids Research, Volume 46, Issue D1, Pages D1003–D1010*, https://doi.org/10.1093/nar/gkx943.

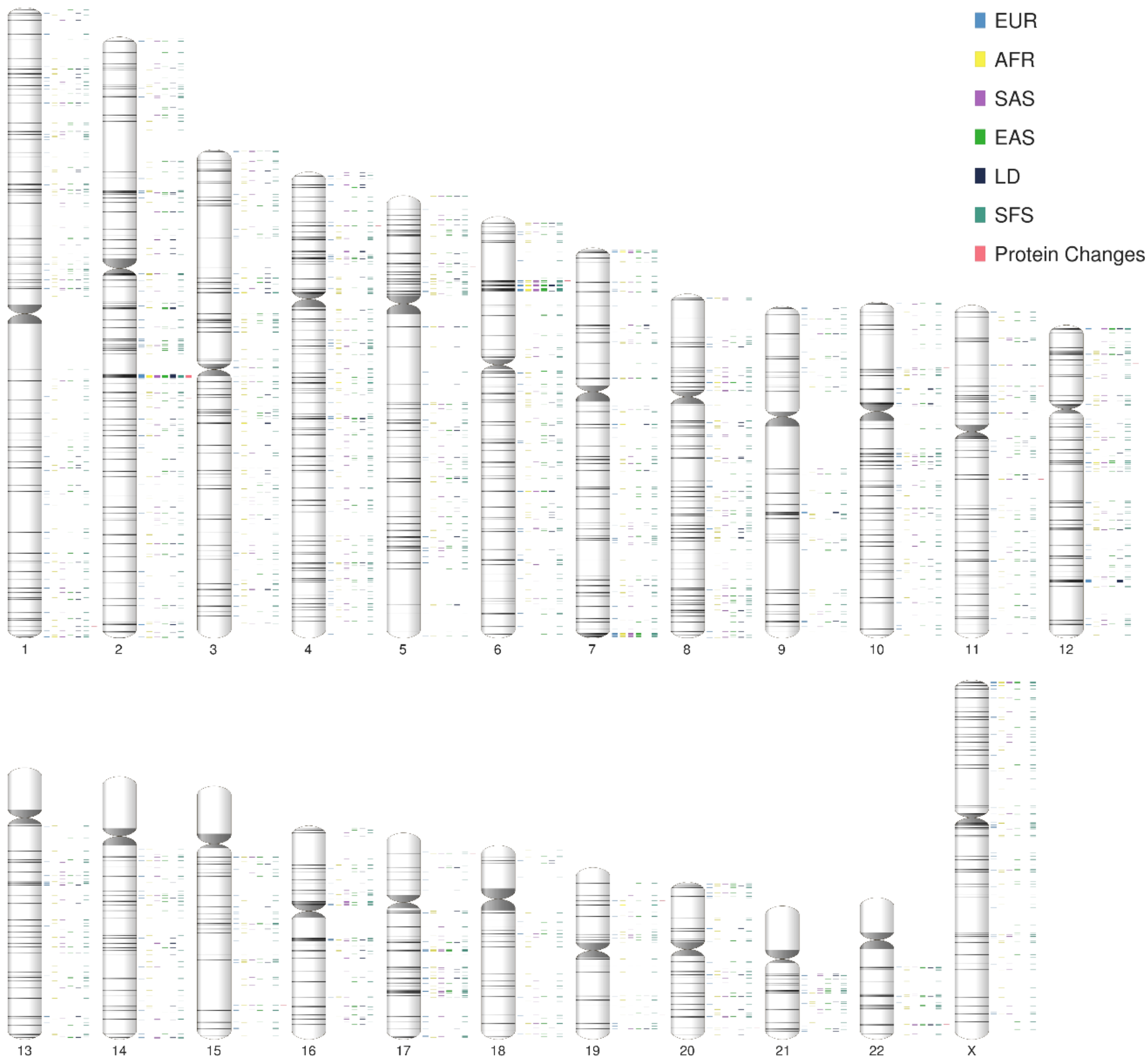

Figure S3. Representation of the candidate regions under selection included in PopHumanScan in a chromosome ideogram. Meta-populations and signature types are color-coded. Meta-populations: ● *African* (AFR), ● *European* (EUR), ● *East-Asian* (EAS), and ● *South-Asian* (SAS). Signature types: ● *Linkage Disequilibrium (LD)*, ● *Site Frequency Spectrum (SFS)*, and ● *Protein Changes.*

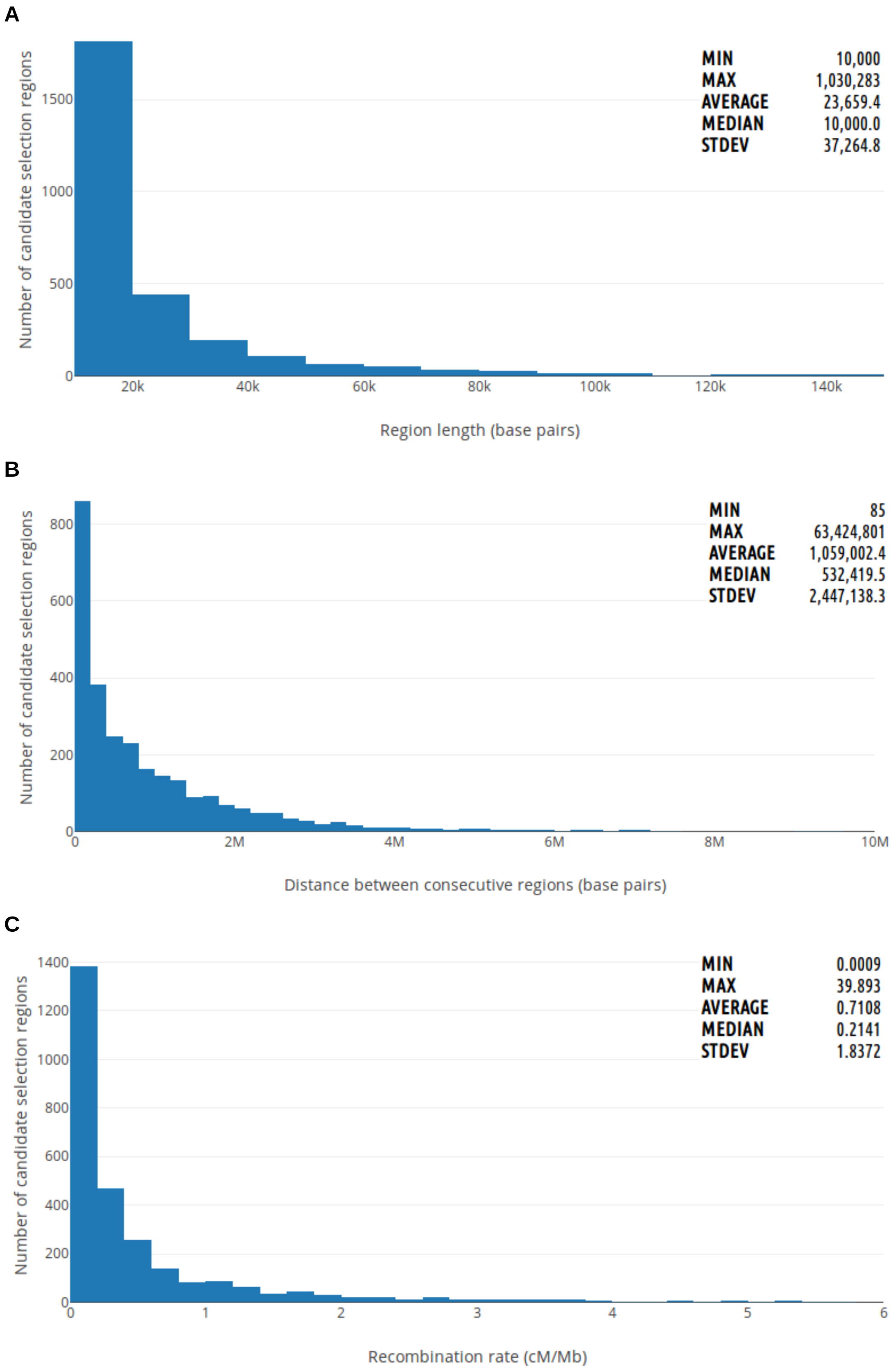

Figure S4. Distribution of the (A) length of candidate regions under selection (bin size = 10kb), (B) distance between consecutive regions (bin size = 200kb), and (C) recombination rate of candidate regions under selection (bin size = 0.2cM/Mb).

A

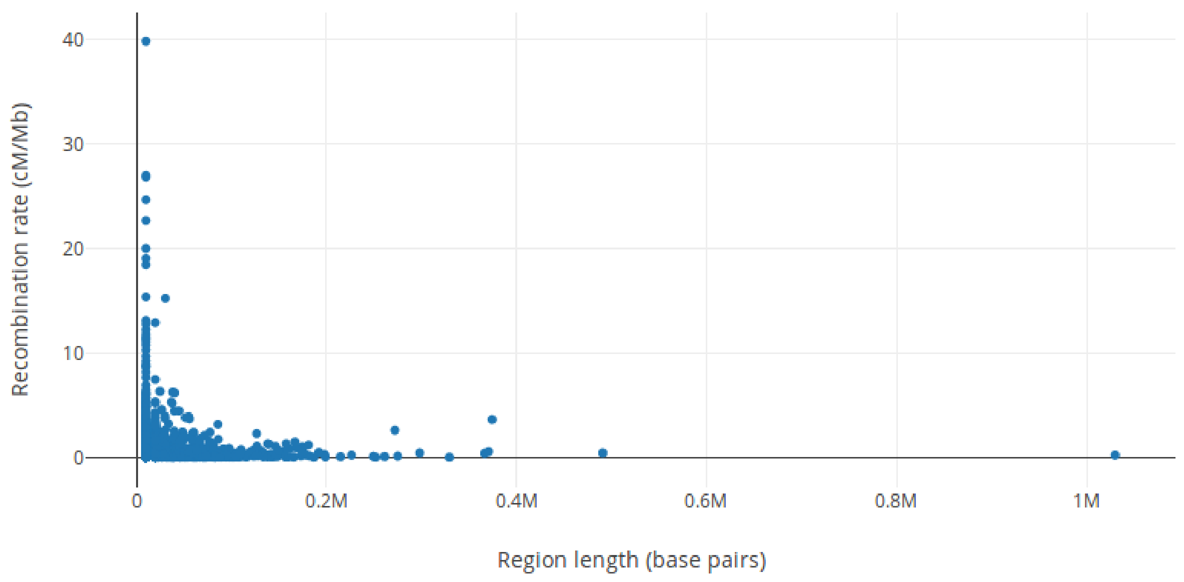

B

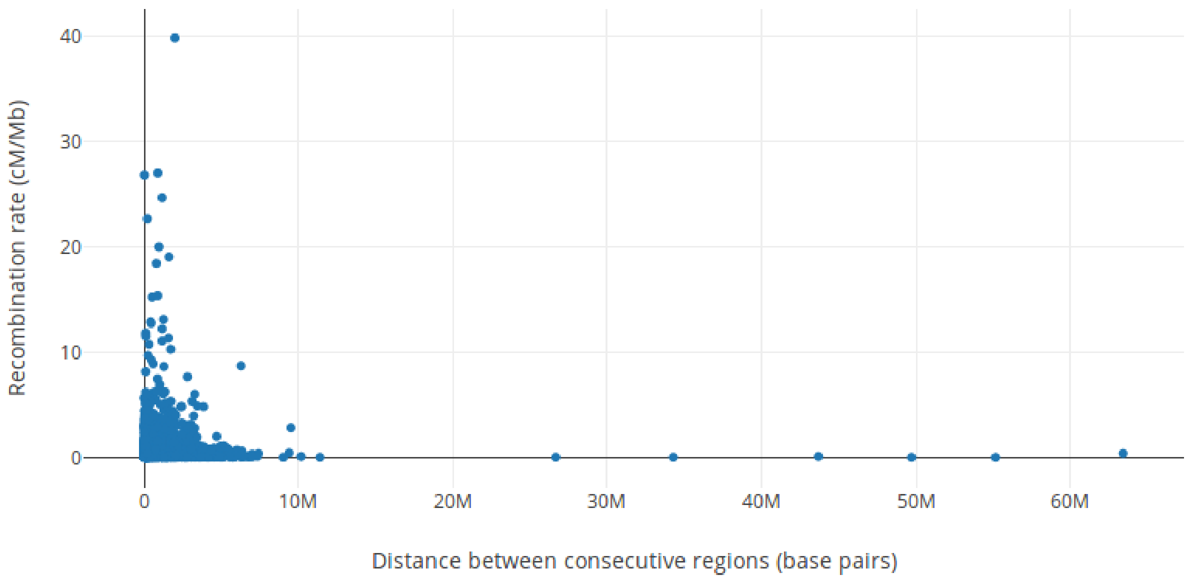

Figure S5. Recombination rate (cM/Mb) as a function of (A) region length (base pairs), and (B) distance between consecutive regions (base pairs).

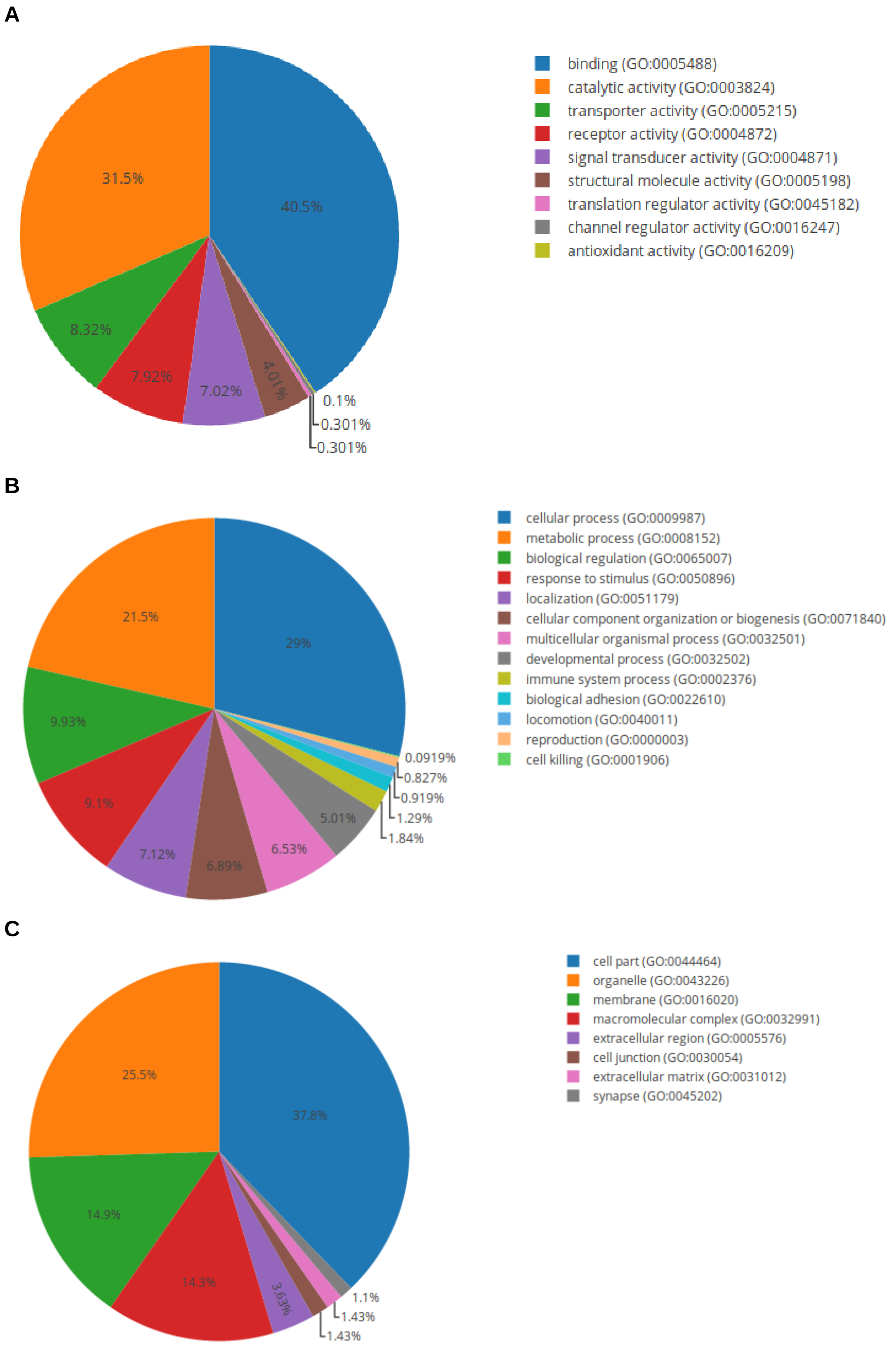

Figure S6. Functional classification of 1,447 GENCODE protein-coding genes overlapping our candidate regions under selection, according to Gene Ontology terms. (A) Molecular Function; (B) Biological Process; (C) Cellular Component.
